# Individual Variation in Intrinsic Neuronal Properties of Nucleus Accumbens Core and Shell Medium Spiny Neurons in Male Rats Prone to Sign- or Goal-Track

**DOI:** 10.1523/ENEURO.0203-25.2025

**Published:** 2025-12-12

**Authors:** Cristina E. María-Ríos, Geoffrey G. Murphy, Jonathan D. Morrow

**Affiliations:** ^1^Neuroscience Graduate Program, University of Michigan, Ann Arbor, Michigan 48109; ^2^Michigan Neuroscience Institute, University of Michigan, Ann Arbor, Michigan 48109; ^3^Departments of Molecular and Integrative Physiology, University of Michigan, Ann Arbor, Michigan 48109; ^4^Psychiatry, University of Michigan, Ann Arbor, Michigan 48109

**Keywords:** electrophysiology, incentive salience, individual differences, intrinsic excitability, pavlovian conditioning, ventral striatum

## Abstract

The “sign-tracking” and “goal-tracking” model of individual variation in associative learning permits the identification of rats with different cue reactivity and predisposition to addiction-like behaviors. Certainly, compared with “goal-trackers” (GTs), “sign-trackers” (STs) show more susceptibility traits such as increased cue-induced “relapse” of drugs of abuse. Different cue- and reward-evoked patterns of activity in the nucleus accumbens (NAc) have been a hallmark of the ST/GT phenotype. However, it is unknown whether differences in the intrinsic neuronal properties of NAc medium spiny neurons (MSNs) in the core and shell subregions are also a physiological correlate of these phenotypes. We performed whole-cell slice electrophysiology in outbred male rats and found that STs exhibited the lowest excitability in the NAc core, with lower number of action potentials and firing frequency as well as a blunted voltage/current relationship curve in response to hyperpolarized potentials in both the NAc core and shell. Although firing properties of shell MSNs did not differ between STs and GTs, intermediate responders that engage in both behaviors showed greater excitability compared with both STs and GTs. These findings suggest that intrinsic excitability in the NAc may contribute to individual differences in the attribution of incentive salience.

## Significance Statement

During associative learning, cues acquire predictive value, but they can also acquire incentive salience, meaning they take on some of the motivational properties of the reward. The propensity to attribute cues with incentive salience varies between individuals, and excessive attribution can lead to maladaptive behaviors. The “sign-and goal-tracking” model allows us to isolate these two properties and disambiguate the neurobiological processes that govern them. This is the first study characterizing passive and active membrane properties of medium spiny neurons in the NAc core and shell of sign-trackers and goal-trackers, as well as intermediate responders. These findings are meant to better inform investigations of the distinct role of the nucleus accumbens in reward learning, particularly in the attribution of incentive salience and addiction predisposition.

## Introduction

The ability to develop adaptive responses to cues signaling food, safety, mating opportunities, and other rewards is essential for survival. The nucleus accumbens (NAc) plays a central role in this learning process. Individual variations in how information about the environment is processed through the NAc could contribute to traits associated with neuropsychiatric disorders including impulsivity, hyperactivity, and cue reactivity (Robinson and Berridge, 1993; Berridge et al., 2009; Flagel and Robinson, 2017). However, linking such behaviorally complex predisposing factors to specific neurobiological processes has proven difficult. Because cues normally acquire both predictive and incentive value together, it is difficult to dissociate these properties and disambiguate the neurobiological processes that govern them. Such a dissociation can be achieved in rats by using pavlovian conditioned approach (PavCA) procedures, which reveal individual differences in associative learning styles known as “sign-tracking” and “goal-tracking” (María-Ríos et al., 2023).

During pavlovian conditioning, “sign-trackers” (STs) reliably approach the reward-paired cue and interact with it (“sign-tracking”), whereas “goal-trackers” (GTs) will direct their behavior away from the cue and toward the site of impending reward delivery (“goal-tracking”). The key difference between these phenotypes is that GTs only use reward-paired cues as predictors, but STs also attribute the cues with incentive salience, meaning the cues become irresistible and rewarding in and of themselves (Berridge et al., 2009). Intermediate responders (IRs) are rats that exhibit a relatively low bias toward sign- or goal-tracking behavior and tend to alternate between both conditioned responses. Compared with GTs, STs are more impulsive (Lovic et al., 2011), have less attentional control (Paolone et al., 2013), and are more susceptible to cue-induced “relapse” of drug self-administration (Saunders and Robinson, 2011; Versaggi et al., 2016), making it a useful model for studying the neurobiological basis of incentive salience attribution and its contribution to disorders like addiction.

Neuronal activity in the NAc seems to be particularly important for individual differences in incentive salience attribution. STs show increased cue-evoked c-*fos* expression in the NAc compared with GTs (Flagel et al., 2011a), and single-unit recordings and in vivo voltammetry have also revealed that STs and GTs exhibit different cue- and reward-evoked patterns of activity and dopamine release in the NAc during pavlovian learning (Flagel et al., 2011b; Gillis and Morrison, 2019). Much research in STs and GTs has focused on dissecting the molecular and neurochemical basis of these differences (for review, see Flagel and Robinson, 2017). However, whether these variations in NAc activity reflect fundamental differences in the intrinsic neuronal excitability of NAc neurons remains unclear.

The NAc consists of two subregions known as the core and shell with distinct anatomical features (Záborszky et al., 1985; Zahm and Heimer, 1990; Heimer et al., 1991; Meredith et al., 1992; Britt et al., 2012) and functional roles in pavlovian learning and motivation (Zahm, 1999; Bassareo et al., 2002; Day and Carelli, 2007; West and Carelli, 2016). GABAergic projection neurons known as medium spiny neurons (MSNs) comprise ∼95% of the total neuronal population in both the core and the shell (Pennartz et al., 1994; Matamales et al., 2009). Therefore, the intrinsic excitability state of MSNs—how sensitive they are to changes in membrane potential caused by input stimuli—may reveal important functional differences between STs, GTs, and IRs in how the NAc integrates and relays reward information (O’Donnell and Grace, 1996; Nicola et al., 2000; Planert et al., 2013; Dorris et al., 2015).

The present study used whole-cell patch-clamp recordings to conduct an electrophysiological characterization of the passive and active membrane properties of MSNs in the NAc core and shell of ST, GT, and IR rats. We hypothesize that MSNs from these three phenotypes will express basal differences in intrinsic excitability and that these will contribute to our understanding of how the NAc, particularly of STs and GTs, differentially encodes predictive and incentive value of appetitive cues.

## Materials and Methods

### Animals

**Figure 1.**
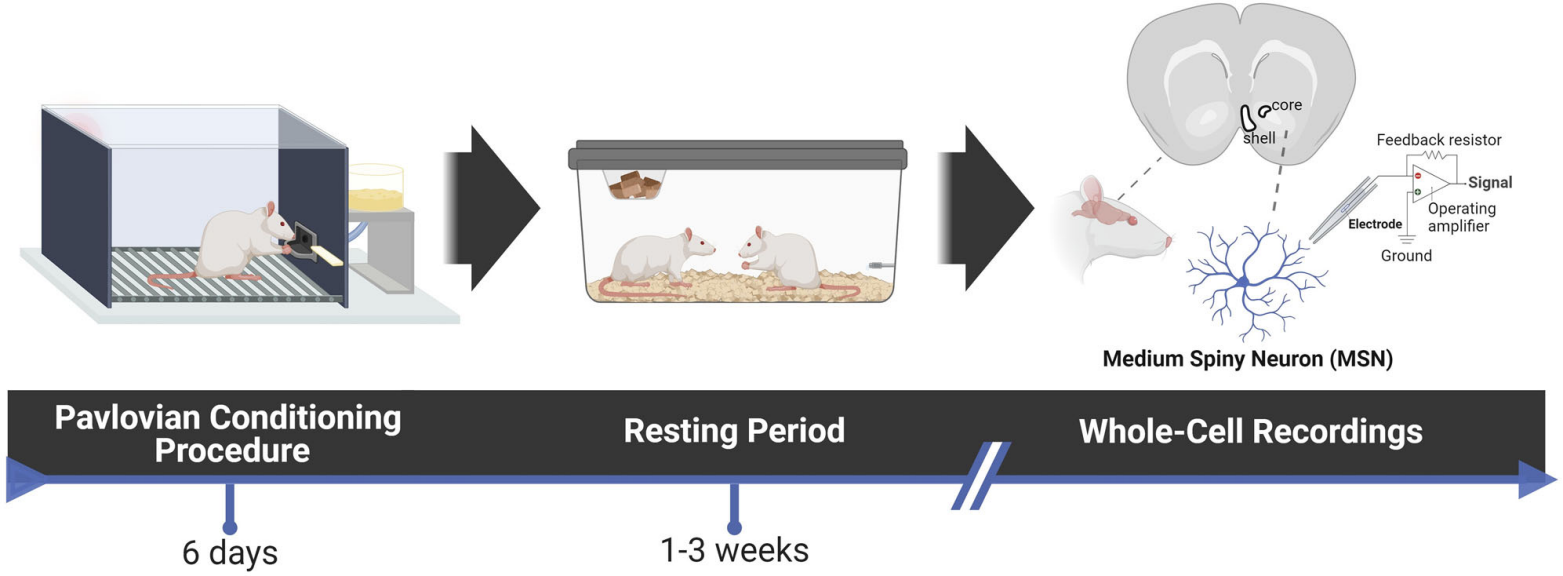
Experimental timeline. Rats underwent a 6 d pavlovian conditioned approach (PavCA) procedure, where a neutral lever cue (CS) was paired with a banana-flavored pellet reward (US) following CS retraction. Each session included 25 CS–US pairings (ITI: 30–60 s). Following a resting period of 1–3 weeks, rats were anesthetized and ex vivo brain slices were prepared. See Extended Data Figure 1-1 for distribution of recovery times across phenotypes. Medium spiny neurons (MSNs) were visualized and whole-cell current-clamp recordings were performed in the nucleus accumbens core and shell. Created with BioRender.

Thirty-one adult male Sprague Dawley rats (aged 8–10 weeks) were purchased from Charles River Laboratories (C72, R04) and were housed in pairs. The rats followed a 12 h light/dark cycle, with *ad libitum* access to food and water throughout the experiment. All rats were acclimatized to the housing colony for at least 2 d prior to handling. Following 7 d of behavioral testing, the rats were allowed a resting period of 1–3 weeks in their home cages before electrophysiological recordings (Fig. 1). All procedures involving animals had received prior approval from the University Committee on the Use and Care of Animals at the University of Michigan, Ann Arbor, MI.

10.1523/ENEURO.0203-25.2025.f1-1 Figure 1-1 *Distribution of recovery times across phenotypes.* Days post-PavCA training to slice preparation are shown for each phenotype. The intended design was a 7-day recovery period; however, because behavioral cohorts were staggered so that each day a rat was ready for recordings, some animals (8 of 31) were recorded beyond 7 days due to equipment availability and scheduling constraints. All phenotypes were represented across the 1–3 week recovery period. Download Figure 1-1, DOCX file.

### Drugs

Isoflurane (Fluriso, VetOne) was administered at 5% via inhalation for inducing anesthesia. Unless otherwise stated, all chemicals were purchased from Tocris Bioscience, Sigma-Aldrich, and Fisher Chemical.

### Behavioral testing apparatus

As previously described (Maria-Rios et al., 2023), 16 operant conditioning chambers with dimensions of 24.1 cm in width, 20.5 cm in depth, and 29.2 cm in height (manufactured by MED Associates) were utilized for behavioral testing. Each chamber was placed within a sound-attenuating cubicle equipped with a ventilation fan for ambient background noise during testing. Every chamber was equipped with a food magazine, a retractable lever (counterbalanced on either the left or right side of the magazine), and a red house light situated on the wall opposite to the magazine. Magazine entries were detected by an infrared sensor, and the levers were calibrated to register deflections in response to a minimum applied weight of 10 g. The inside of the lever mechanism featured a mounted LED to illuminate the slot through which the lever extended each time it protruded into the chamber. ABET II Software (Lafayette Instrument) was used to automatically record the number and latency of lever presses and magazine entries.

### Behavioral testing procedure

**Figure 2.**
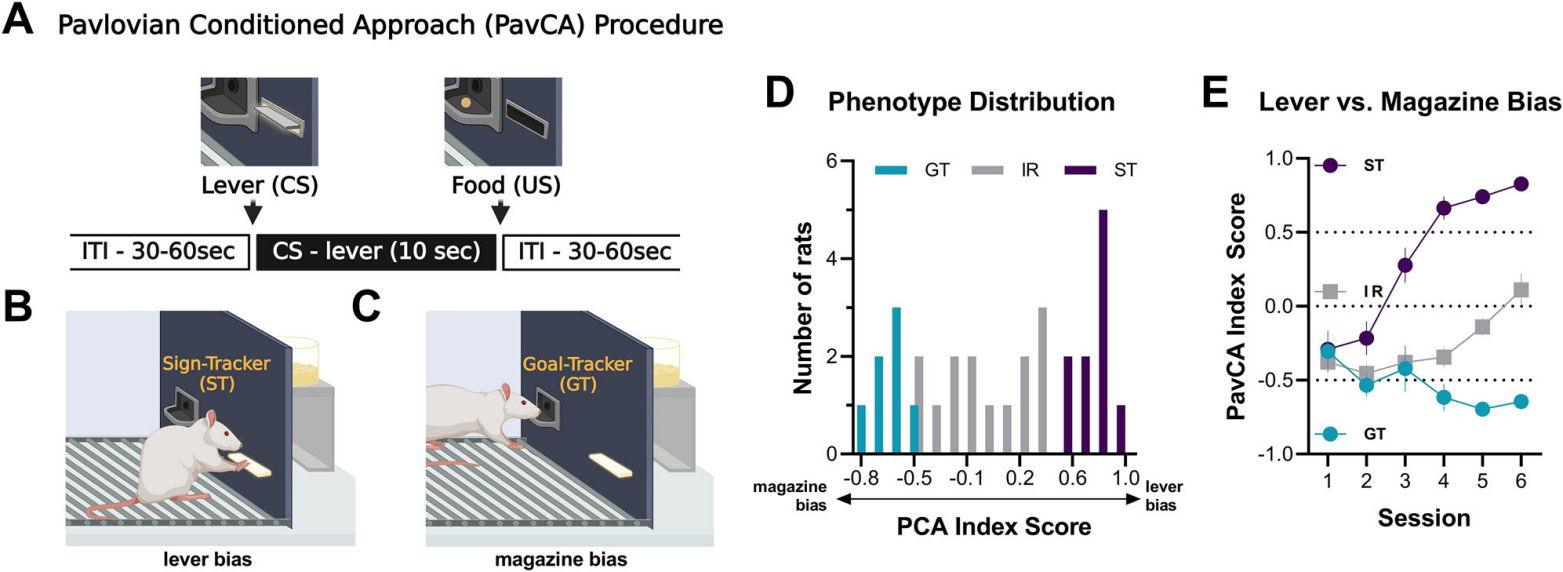
Classification of pavlovian conditioned approach phenotypes. Rats were classified as sign-trackers (ST; *n* = 10), goal-trackers (GT; *n* = 7), or intermediate responders (IR; *n* = 14) based on their lever and magazine bias during PavCA. ***A***, Each PavCA trial involved the extension of a lever cue (CS) followed by food pellet delivery (US) after 10 s (intertrial interval: 30–60 s). ***B***, STs approach the lever during the CS, despite no need for interaction for reward. ***C***, GTs approach the magazine, the site of food delivery, in response to the CS. ***D***, Distribution of ST (score ≥ 0.5), GT (score ≤ −0.5), and IR (−0.5 < score < 0.5) phenotypes. ***E***, PavCA index scores for ST, GT, and IR groups across six training sessions, illustrating the divergence of phenotypes over time. Data are presented as mean ± SEM. Diagrams created with BioRender.

Pavlovian conditioning was performed as previously described (María-Ríos et al., 2023). For 2 d prior to the start of training, all rats were habituated to the handler and the food reward. Rats were handled individually and were given banana-flavored pellets (45 mg; Bio-Serv) in their home cages. On the third day, the rats were subjected to one pretraining session in the test chambers. Twenty-five food pellets were delivered on a variable time (VT) 30 s schedule (i.e., one pellet was delivered on average every 30 s, but varied 0–60 s). During this session, the lever remained retracted. Following pretraining, all rats underwent six daily sessions of behavioral training (Fig. 2*A*–*C*). Each trial consisted of a presentation of the illuminated lever (conditioned stimulus, CS) into the chamber for 10 s on a VT 45 s schedule (i.e., time randomly varied 30–60 s between CS presentations). Immediately after retraction of the lever, there was a response-independent delivery of one pellet into the magazine (unconditioned stimulus, US). The beginning of the next intertrial interval (ITI) began once both the lever and the pellet had been presented, and each test session consisted of 25 trials of CS–US pairings. All rats consumed all the pellets that were delivered. Rats were not food deprived at any point during experimentation.

### Electrophysiology

#### Slice preparation

After a 1–3 week resting period following the final day of PavCA training, slice preparation was performed as previously described (Maria-Rios et al., 2023). All rats were killed by decapitation after inducing anesthesia with an isoflurane machine (Kent Scientific). Following rapid brain dissection, the brain was glued on a platform, which was then submerged in an ice-cold oxygenated (95% O_2_/5% CO_2_) cutting solution containing the following (in mM): 206 sucrose, 10 d-glucose, 1.25 NaH_2_PO_4_, 26 NaHCO_3_, 2 KCl, 0.4 sodium ascorbic acid, 2 MgSO_4_, 1 CaCl_2_, and 1 MgCl_2_. A mid-sagittal cut was made to divide the two hemispheres, and 300 µm coronal brain slices were cut using a vibrating blade microtome (Leica VT1200). The brain slices were transferred to a holding chamber with oxygenated aCSF containing the following (in mM): 119 NaCl, 2.5 KCl, 1 NaH_2_PO_4_, 26.2 NaHCO_3_, 11 d-glucose, 1 sodium ascorbic acid, 1.3 MgSO_4_, and 2.5 CaCl_2_ (∼295 mOsm, pH 7.2–7.3) at 37°C for 20 min and then room temperature for at least 40 min of rest for a total of 1 h. The slices were kept submerged in oxygenated aCSF in a holding chamber at room temperature for up to 7–8 h following slicing.

#### Electrophysiological recordings

All electrophysiological procedures were performed as previously described (Maria-Rios et al., 2023). After allowing the slices to rest for at least 1 h, individual slices were transferred to a recording chamber perfused with oxygenated aCSF (32°C) containing 100 µM of the GABA_A_ receptor antagonist picrotoxin and 5 mM kynurenic acid to block glutamatergic transmission. Core and medial shell recordings from the NAc were made in the same slices obtained between +1.00 and +1.70 mm anterior from bregma (Paxinos and Franklin, 2019). The core and shell were distinguished based on their anatomical boundaries: the NAc core was identified as the region adjacent to the anterior commissure, while the medial shell was located ventromedial to the core, delineated by differences in cytoarchitecture and proximity to the lateral septum. Infrared differential interference contrast (IR-DIC) optics (microscope, Olympus BX51; camera, Dage-MTI) were used to visualize cells. Whole-cell current-clamp recordings were performed using borosilicate glass pipettes (O.D. 1.5 mm, I.D. 0.86 mm; Sutter Instrument) with a 4–7 MΩ open tip resistance filled with a potassium gluconate-based internal solution containing the following (in mM): 122 K-gluconate, 20 HEPES, 0.4 EGTA, 2.8 NaCl, and 2 Mg^2+^ATP/0.3 Na_2_GTP (∼280 mOsm, pH adjusted to 7.2 with KOH). MSNs were identified based on morphology (medium-sized soma) as well as a hyperpolarized resting potential between −70 and −90 mV and inward rectification. Patched neurons were excluded based on a resting potential out of the desired range, characteristics of fast-spiking interneurons, and irregular firing patterns. All recordings were obtained using the MultiClamp 700B (Molecular Devices) amplifier and Digidata 1550A (Molecular Devices) digitizer. Data were filtered at 2 kHz, digitized at 10 kHz, and collected and analyzed using pClamp 10.0 software (Molecular Devices).

For whole-cell recordings, a membrane seal with a resistance >1 GΩ was achieved prior to breaking into the cell. Membrane capacitance (*C*_m_) and series resistance (*R*_s_) were recorded 1 min after breaking in and were compensated under voltage clamp. *R*_s_ was recorded in voltage clamp with an average of 28 ± 6 MΩ upon entry and 26 ± 6 MΩ once the recording was finished for STs, 28 ± 7 MΩ and 27 ± 7 MΩ for GTs, and 30 ± 7 MΩ and 26 ± 7 MΩ for IRs (mean ± SD). Firing properties were recorded under current-clamp, and input resistance (*R*_i_) was monitored online during each sweep with a −100 pA, 25 ms current injection separated by 100 ms from the current injection step protocols. The average *R*_i_ across all sweeps is reported, and only cells with an *R*_i_ that remained stable (Δ < 20%) were included in the analysis (STs, *n* = 42; GTs, *n* = 40; IRs, *n* = 46). To assess firing properties, neurons were subjected to two recording protocols from their resting membrane potential (RMP). For spike number, spike frequency, voltage/current relationships, and sag ratios, neurons were subjected once to a step protocol consisting of 500 ms current injections starting from −500 to +500 pA in 25 pA increments with each sweep separated by 4 s. The RMP was calculated as the average voltage across all sweeps at 5 ms. Spike number was determined by counting individual action potentials at each level of current injection. Firing frequency (Hz) was calculated by averaging the frequency between consecutive spikes for a given current injection. If a neuron exhibited depolarization block, data for that cell were included up to the last current injection before the block occurred. Steady-state voltage responses were measured 200 ms after stimulus onset for each subthreshold current injection step. Sag ratios were calculated as the ratio of the peak voltage at the most hyperpolarized current injection (−500 pA) to the steady-state response. A ratio of 1.0 indicated no sag, with larger ratios corresponding to greater sag. The voltage/current relationship was assessed by measuring the difference between the steady-state voltage and the baseline voltage recorded 1 ms before stimulus onset.

To examine single action potential (AP) properties, neurons received 5 ms current injections in 25 pA increments, with each sweep separated by 4 s, until a single AP was elicited. The minimal current required to evoke an AP was defined as the current threshold (pA). AP threshold (mV) was determined as the voltage at the AP inflection point relative to 0 mV. The difference between RMP and AP threshold (Δ RMP/AP threshold, mV) was calculated by subtracting the AP threshold from the RMP. AP amplitude (mV) was measured as the voltage at the peak of the AP overshoot relative to 0 mV, and the difference between RMP and AP amplitude (Δ RMP/AP amplitude, mV) was also calculated. Similarly, the difference between AP threshold and AP amplitude (Δ AP threshold/AP amplitude, mV) was determined. AP half-width (ms) was defined as the duration of the AP at half of its peak amplitude.

### Experimental design and statistical analysis

#### Behavioral studies

The behavioral response in the pavlovian conditioned approach (PavCA) procedure was scored using an index that integrates the number, latency, and probability of lever presses (sign-tracking conditioned response) and magazine entries (goal-tracking conditioned response) during CS presentations within a session (Meyer et al., 2012). In brief, we averaged the response bias (i.e., number of lever presses and magazine entries for a session; [lever presses − magazine entries] / [lever presses + magazine entries]), latency score (i.e., average latency to perform a lever press or magazine entry during a session; [magazine entry latency − lever press latency] / 10), and probability difference (i.e., proportion of lever presses or magazine entries; lever press probability − magazine entry probability). The index score ranges from +1.0 (absolute sign-tracking) to −1.0 (absolute goal-tracking), with 0 representing no bias. The average PavCA index scores of Sessions 5–6 were used to classify rats as STs (score ≥ 0.5), GTs (score ≤ −0.5), and IRs (−0.5 < score < 0.5). Out of a total of 31 rats, 10 were classified as STs, 7 were GTs, and 14 were IRs (Fig. 2*B*–*D*).

#### Electrophysiology studies

A total of 128 cells from 31 rats were included in the analysis. The groups were divided as follows: STs group had a total of 10 rats, from which 23 cells were recorded in the core of 10 rats (1–2 slices/1–5 cells per rat) and 19 cells in the shell of 9 of the rats (1–2 slices/1–3 cells per rat); GTs group had a total of 7 rats, from which 17 cells were recorded in the core of 7 rats (1–2 slices/2–4 cells per rat) and 23 cells in the shell of 7 rats (1–2 slices/2–5 cells per rat); IRs group had a total of 14 rats, from which 31 cells were recorded in the core of 14 rats (1–2 slices/1–5 cells per rat) and 15 cells in the shell of 10 of the rats (1 slice/1–3 cells per rat).

For the analyses of sag ratio and voltage/current relationship curve, a total of 8 cells from STs (Core: *n* = 4, Shell: *n* = 4) and 17 cells from IRs (Core: *n* = 13, Shell: *n* = 4) were excluded. These cells underwent a step protocol ranging from −200 to +500 pA instead of the standard −500 to +500 pA and were removed to maintain consistency in the hyperpolarized current injection analysis. Additionally, the single action potential protocol was not performed for 3 ST core cells, 1 GT core cell, 4 IR core cells, and 2 IR shell cells.

Electrophysiological data were analyzed offline using Clampfit 10.7 (Molecular Devices). Statistical analyses were conducted using GraphPad Prism 8 (Dotmatics) and SPSS Statistics (IBM). RMP, *C*_m_, *R*_i_, sag ratio, current to threshold, AP threshold, ΔRMP/AP threshold, AP amplitude, ΔRMP/AP amplitude, ΔAP threshold/AP amplitude, and AP half-width were analyzed separately for the core and shell using one-way ANOVA to assess group differences between STs, GTs, and IRs followed by Tukey's post hoc test for multiple comparisons (significance threshold: *p* < 0.05).

Number of spikes, spike firing frequency, and voltage/current (*V*/*I*) relationship curves were analyzed using repeated-measures linear mixed-effects model (REML), with significance set at *p* < 0.05. Electrophysiological properties were analyzed primarily at the cell level to capture within-animal variability. To control for nonindependence, we also computed per-animal means by averaging across all recorded cells within each region (core, shell) for each rat. To assess phenotype differences within each subregion, fixed effects were defined for phenotype (STs, GTs, IRs), current injection (*V*/*I*: −500 to 0 pA, 0 to +100 pA; AP: +25 to +500 pA), and phenotype × current injection. Planned comparisons using mixed-effects model (REML) were conducted based on Tukey's post hoc test to obtain statistical values (F/P) for STs versus GTs, STs versus IRs, and GTs versus IRs. For subregional analysis of *V*/*I* relationships, number of spikes, and firing frequency, repeated-measures mixed-effects model (REML) were performed separately within each phenotype (STs, GTs, IRs). Fixed effects were defined for subregion (core, shell), current injection (*V*/*I*: −500 to 0 pA, 0 to +100 pA; AP: +25 to +500 pA), and subregion × current injection.

Exploratory regressions were conducted to examine whether PavCA index (mean of Sessions 5–6) predicted intrinsic membrane properties. For each animal, values were averaged across recorded cells within a given region (core or shell) to yield one data point per animal per property. Linear regressions were then performed in GraphPad Prism 10 with PavCA index as the predictor and either RMP, *R*_i_, or spike number at 500 pA as the dependent variable.

## Results

### Following a PavCA procedure, rats were classified as STs, GTs, or IRs based on their lever and magazine response bias

To identify rats with ST, GT, and IR behavioral phenotypes in an outbred population, all rats underwent 6 d of PavCA training with a lever cue (CS) paired with the response-independent delivery of a banana-flavored food pellet into a magazine (US; Fig. 2*A*–*C*). Based on their PavCA index score on Sessions 5–6, rats were classified as either STs (score ≥ 0.5; *n* = 10), GTs (score ≤ −0.5; *n* = 7), or intermediate responders (−0.5 < score < 0.5; *n* = 14; Fig. 2*D*). PavCA index scores clearly diverged across sessions, with STs increasingly biased toward lever-oriented behavior and GTs biased toward magazine-oriented behaviors, while IRs exhibited both responses (Fig. 2*E*).

**Figure 3.**
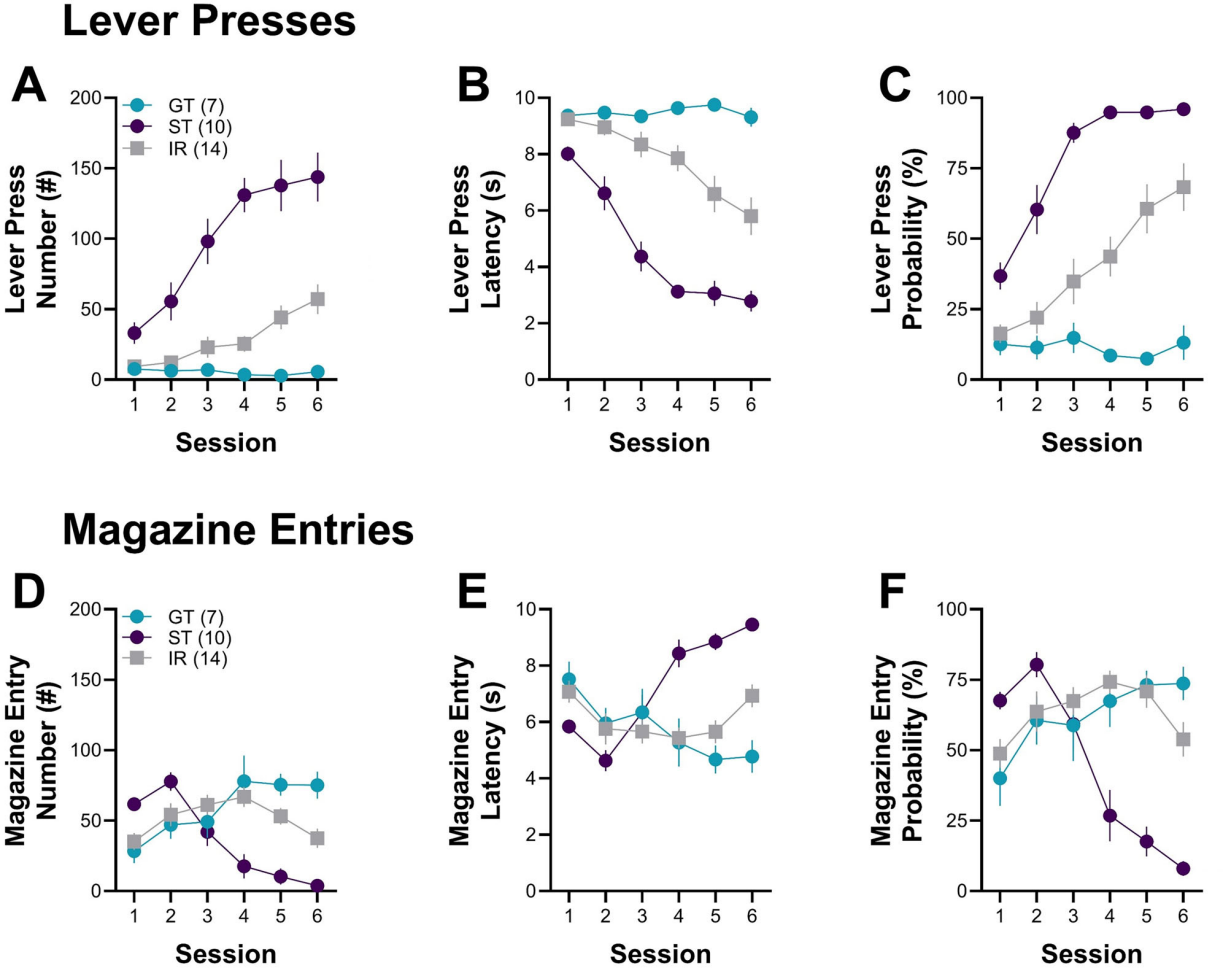
Behavioral differences in lever pressing and magazine entries during PavCA. STs and GTs differed in the number, latency, and probability of lever presses and magazine entries across six PavCA sessions. ***A***, Lever press number, ***B***, latency, and ***C***, probability over sessions for STs, GTs, and IRs. ***D***, Magazine entry number, ***E***, latency, and ***F***, probability across sessions. STs primarily engaged in lever-oriented behaviors, whereas GTs displayed magazine-oriented behaviors. IRs showed intermediate responding between the two groups. Data are presented as mean ± SEM.

Behavioral measures confirmed the distinct response patterns across phenotypes. STs exhibited higher lever press number, shorter lever press latency, and greater probability of lever pressing across training compared with GTs, with IRs showing intermediate levels (Fig. 3*A*–*C*). In contrast, GTs displayed more frequent, faster, and more probable magazine entries, whereas STs showed fewer and slower entries; IRs again exhibited intermediate responding (Fig. 3*D*–*F*). These descriptive patterns align with previous reports demonstrating that STs readily approach and interact with reward-predictive cues, whereas GTs direct their behavior toward the location of reward delivery (Flagel et al., 2009; Maria-Rios et al., 2023). The inclusion of multiple behavioral parameters allows for a more comprehensive characterization of response acquisition, highlighting the distinct learning strategies employed by each phenotype.

### Passive membrane properties of NAc MSNs in the core and shell subregions of STs, GTs, and IRs rats

**Figure 4.**
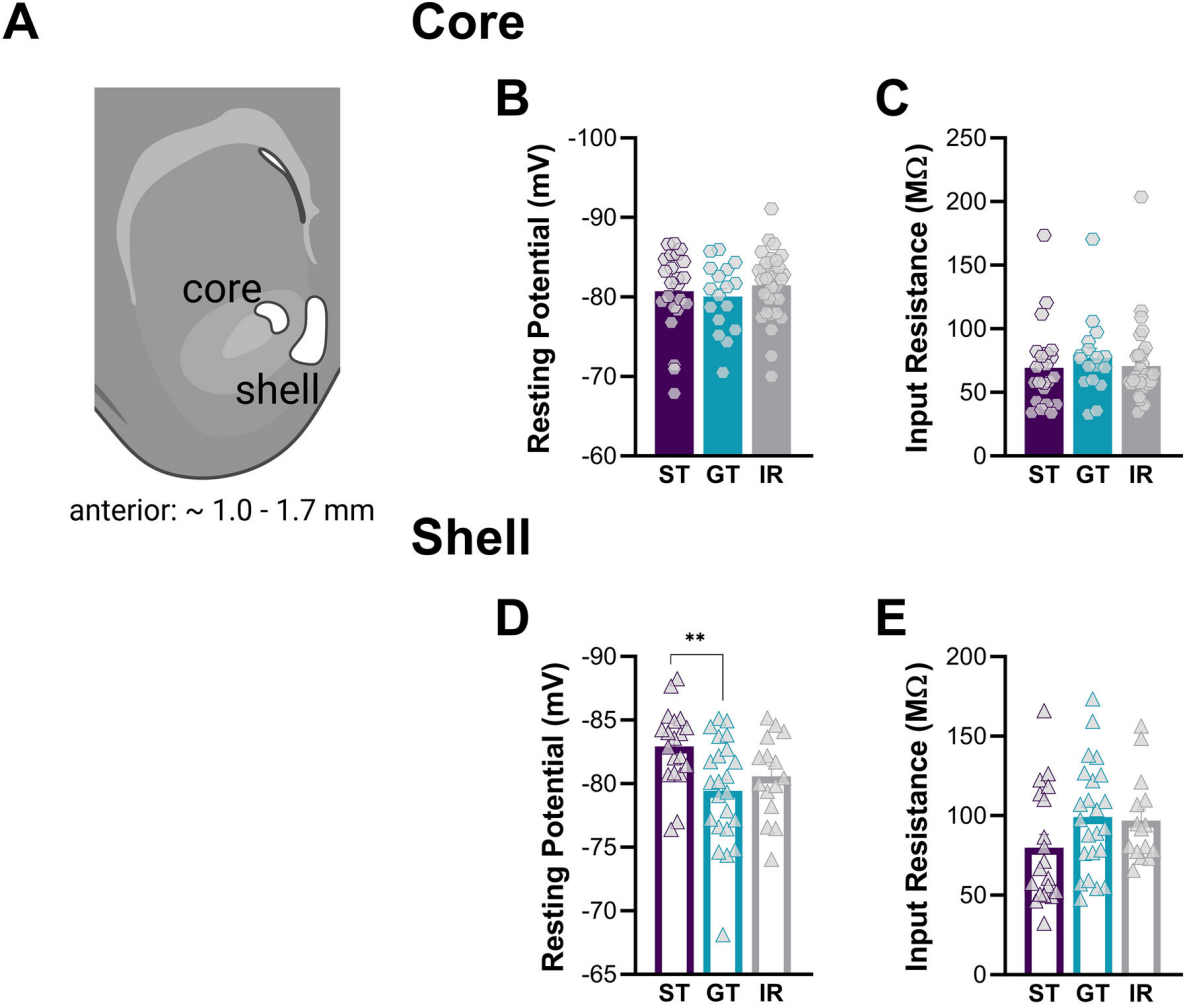
Passive membrane properties of NAc MSNs in ST, GT, and IR rats. ***A***, Diagram showing the NAc core and shell subregions where whole-cell recordings were made. ***B***, Resting potential and ***C***, input resistance in the NAc core showed no significant differences between STs, GTs, and IRs. ***D***, In the NAc shell, STs had a more hyperpolarized resting membrane potential compared with GTs. ***E***, No differences in input resistance were observed across groups in the NAc shell. Significance for Tukey's post hoc test is shown as **p < 0.01. Data are presented as mean ± SEM. See Extended Data Figure 4-1 for per-animal mean electrophysiological properties of NAc MSNs and Extended Data Figure 4-2 for exploratory regressions between PavCA index and intrinsic properties.

To assess whether STs, GTs, and IRs exhibit differences in intrinsic excitability, we examined the passive membrane properties of MSNs in the NAc core and shell (Fig. 4*A*). Whole-cell patch-clamp recordings were performed in rat brain slices following a 1–3 week resting period from the last PavCA training session (Fig. 1). This resting period was intended to allow training-induced neuronal changes to return to baseline so that our measurements would reflect innate differences in intrinsic excitability across the three phenotypes. Animals from all phenotypes were represented across this range, with no systematic differences in recovery time between groups (Extended Data Fig. 1-1). No significant main effects of phenotype were observed in the NAc core for resting membrane potential (RMP; *F*_(2,68)_ = 0.55, *p* = 0.58) or input resistance (*F*_(2,68)_ = 0.33, *p* = 0.72; Fig. 4*B*,*C*). However, in the NAc shell, a significant main effect of phenotype was detected for RMP (*F*_(2,54)_ = 4.98, *p* = 0.010). Post hoc analysis indicated that STs exhibited a significantly more hyperpolarized RMP compared with GTs (*p* = 0.0079), while IRs did not differ significantly from the other groups (Fig. 4*D*). Planned per-animal comparisons also supported this finding, with STs showing a more hyperpolarized RMP in the shell than GTs (*t*_(14)_ = 2.54, *p* = 0.0278; Extended Data Fig. 4-1*F*). Input resistance remained nonsignificant across phenotypes (*F*_(2,54)_ = 1.96, *p* = 0.15; Fig. 4*E*). These findings suggest that differences in passive properties between phenotypes may be more prominent in the NAc shell than in the core, with STs exhibiting a more hyperpolarized RMP. This could indicate a lower baseline excitability in ST MSNs, potentially contributing to differences in how reward-related cues are processed and attributed with incentive salience.

10.1523/ENEURO.0203-25.2025.f4-1 Figure 4-1 *Per-animal mean electrophysiological properties of NAc MSNs.* Passive and active membrane properties were averaged per animal (one point per rat) and plotted for the NAc core and shell. For passive properties, no overall phenotype effects were observed, but a planned comparison confirmed that STs exhibited significantly more hyperpolarized RMP in the shell compared to GTs. For active properties, mixed-effects analyses did not reveal overall differences across phenotypes, but a planned comparison showed a significant ST vs GT interaction in the core for action potential number across current steps, in line with the cell-level findings. Core vs shell comparisons further revealed significant excitability differences for STs and IRs but not GTs. Significance for mixed-effects model planned comparisons and planned unpaired t-test is shown as *p < 0.05, ****p < 0.0001. Data are presented as mean ± S.E.M. Download Figure 4-1, TIF file.

10.1523/ENEURO.0203-25.2025.f4-2 Figure 4-2 *Exploratory regressions between PavCA index and intrinsic properties.* (A–C) NAc core: PavCA index vs number of spikes at 500 pA (A), resting membrane potential (B), and input resistance (C). (D–F) NAc shell: PavCA index vs number of spikes at 500 pA (D), resting membrane potential (E), and input resistance (F). Each point represents one animal; solid lines show linear regression with 95% CI (dotted). Regression results (R², p) are shown on each panel. Download Figure 4-2, TIF file.

To complement the group-level comparisons, we also conducted exploratory regressions using PavCA index as a continuous measure (Extended Data Fig. 4-2). In line with the ANOVA results, PavCA index was not associated with RMP or input resistance in the core. However, in the shell, PavCA index showed a negative association with RMP (*R*^2^ = 0.22, *p* = 0.015), consistent with the more hyperpolarized resting potentials observed in STs at the group level (Extended Data Fig. 4-2*E*). Input resistance in the shell was not significantly related to PavCA index. These exploratory findings provide convergent evidence that differences in baseline membrane potential are linked to sign-tracking behavior.

### NAc MSNs in the core and shell of STs exhibit lower hyperpolarization than GTs and IRs in response to negative current inputs

**Figure 5.**
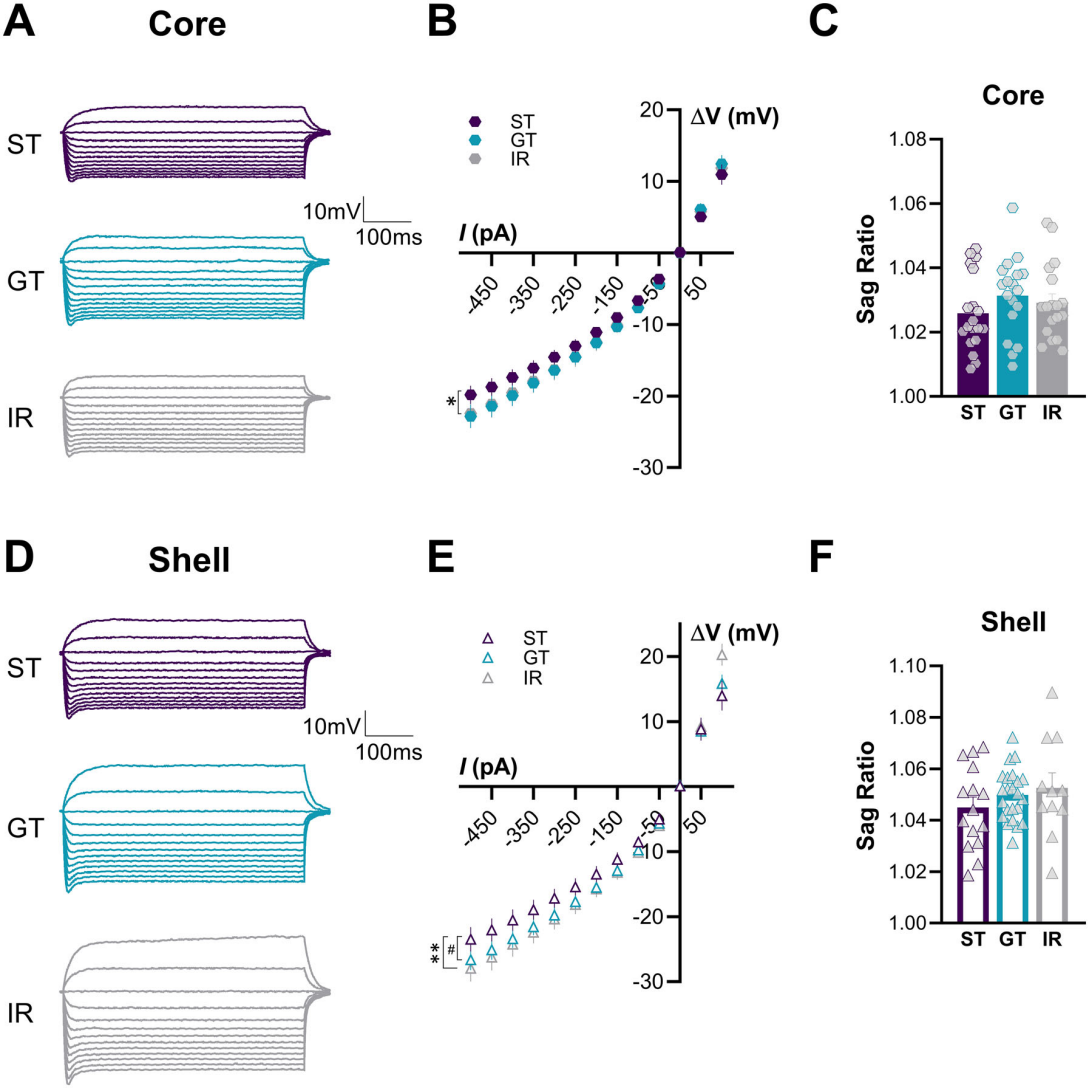
Differential hyperpolarizing responses of NAc MSNs in STs, GTs, and IR. ***A***, ***D***, Representative voltage traces from current-clamp recordings of MSNs in the NAc core and shell from STs, GTs, and IRs. ***B***, ***E***, Voltage/current relationship: STs exhibited significantly lower hyperpolarization in response to negative current injections compared with GTs in the core and to both GTs and IRs the NAc shell. No differences were found between phenotypes in response to depolarizing current injections. ***C***, ***F***, Sag ratio: No significant differences were observed between phenotypes in the sag ratio in the NAc core or shell. Significance for mixed-effects model planned comparisons (phenotype × current interaction) is shown as *p < 0.05, **p < 0.01, ^#^*p* = 0.05. Data are presented as mean ± SEM.

We next examined whether active intrinsic excitability properties differed across phenotypes. To assess this, MSNs in the NAc core and shell were subjected to a current injection step protocol ranging from −500 to +500 pA in 25 pA increments (Fig. 5*A*,*D*). We first analyzed the voltage/current (*V*/*I*) relationship for hyperpolarizing currents (−500 to 0 pA) to determine whether the neurons' responses to negative current injections varied by phenotype. Repeated-measures mixed-effects analyses across all three phenotypes did not reveal a significant overall effect of phenotype or interaction in the core or shell. However, planned pairwise comparisons showed a phenotype × current interaction, highlighting phenotype-specific differences. In the core, STs exhibited significantly lower hyperpolarization compared with GTs (*F*_(9,315)_ = 2.098, *p* = 0.0294) across negative current steps (Fig. 5*B*). In the shell, STs also showed reduced hyperpolarization relative to GTs (trend: *F*_(9,324)_ = 1.900, *p* = 0.0513) and significantly less hyperpolarization than IRs (*F*_(9,216)_ = 2.862, *p* = 0.0033; Fig. 5*E*). These planned comparisons suggest that ST MSNs are less responsive to hyperpolarizing input, which could influence how reward-related information is processed in the NAc. In contrast, when examining responses to depolarizing current injections (0–100 pA), no significant interaction was found in either the core (*p* = 0.7100) or shell (*p* = 0.0684), suggesting that responses to positive currents were comparable across phenotypes. This suggests that while ST MSNs differ in their hyperpolarization response, their response to positive current remain largely unchanged relative to GTs and IRs.

To further examine differences in neuronal excitability, we analyzed the sag response, a measure of how neurons recover from hyperpolarization and regulate their membrane potential following inhibitory input (Pape, 1996; Robinson and Siegelbaum, 2003). The sag ratio was obtained by dividing the peak voltage response to −500 pA over the steady-state response 200 ms from stimulus onset. No significant differences were observed across phenotypes (Core: *F*_(2,52)_ = 1.01, *p* = 0.37; Shell: *F*_(2,46)_ = 0.97, *p* = 0.39; Fig. 5*C*,*F*). This suggests that MSNs across phenotypes exhibit similar capacities to stabilize their membrane potential after hyperpolarizing inputs.

Overall, across all STs (*F*_(12,381)_ = 2.566, *p* = 0.0028), GTs (*F*_(12,463)_ = 3.565, *p* < 0.0001), and IRs (*F*_(12,322)_ = 8.035, *p* < 0.0001) phenotypes, NAc MSNs in the shell exhibited significantly greater excitability than core MSNs (subregion × current interaction), as both hyperpolarizing and depolarizing current injections (−500 to +100 pA) consistently induced a larger change in membrane potential in the shell. Importantly, a subregion × current interaction was also confirmed in per-animal repeated-measures analyses for STs (*F*_(12,144)_ = 2.697, *p* = 0.0026), GTs (*F*_(12,144)_ = 5.632, *p* < 0.0001), and IRs (*F*_(12,143)_ = 4.003, *p* < 0.0001), further supporting greater shell excitability independent of unit of analysis (data from Extended Data Fig. 4-1*D*,*I*). This aligns with prior research demonstrating that the NAc shell is generally more excitable than the core (Kourrich et al., 2007; Maria-Rios et al., 2023), possibly reflecting differences in synaptic input integration and output connectivity between these subregions.

### NAc MSNs in the core and shell of STs, GTs, and IRs exhibit distinct firing properties

**Figure 6.**
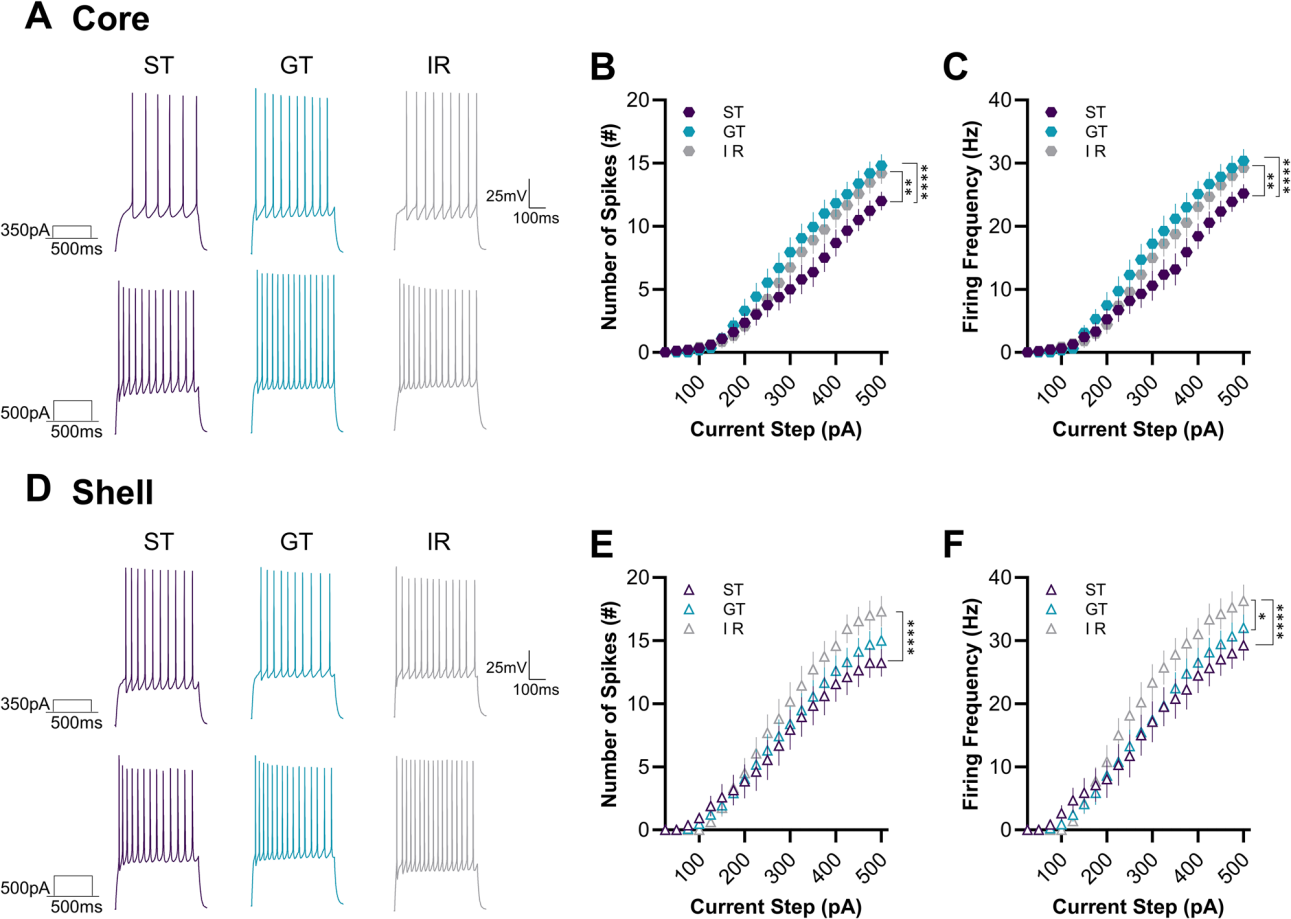
Distinct firing properties of NAc MSNs in STs, GTs, and IRs. ***A***, ***D***, Representative current-clamp traces from MSNs in the NAc core and shell following 350 pA (top) and 500 pA (bottom) current injections in STs, GTs, and IRs. ***B***, ***C***, In the NAc core, STs exhibited the lowest number of spikes and firing frequency, while GTs and IRs did not differ from one another. ***E***, ***F***, In the NAc shell, IRs had significantly higher spikes than STs and greater firing frequency compared with both STs and GTs, with no differences between STs and GTs. Significance for mixed-effects model planned comparisons (phenotype × current interaction) is shown as *p < 0.05, **p < 0.01, ****p < 0.0001. Data are presented as mean ± SE.M.

To further characterize the intrinsic excitability of MSNs from the core and shell of STs, GTs, and IRs, we examined their firing properties in response to current injection steps ranging from +25 to +500 pA in 25 pA increments (Fig. 6*A*,*D*). In the NAc core, repeated-measures mixed-effects analyses revealed a significant phenotype × current interaction for spike number (*F*_(38,1248)_ = 2.027, *p* = 0.0003) and frequency (*F*_(38,1248)_ = 2.037, *p* = 0.0002). Planned pairwise comparisons indicated that STs MSNs fired significantly fewer spikes than both GTs (*F*_(19,712)_ = 3.561, *p* < 0.0001) and IRs (*F*_(19,947)_ = 2.248, *p* = 0.0017) across current steps, whereas GTs and IRs did not differ from one another (*p* = 0.9152; Fig. 6*B*). Firing frequency analyses showed the same pattern, with STs exhibiting lower firing rates than both GTs (*p* < 0.0001) and IRs (*p* = 0.0011), while GTs and IRs did not differ (*p* = 0.9038; Fig. 6*C*). Planned per-animal analyses further revealed a significant ST versus GT interaction in the core for action potential number across current steps, consistent with the cell-level findings (*F*_(19,266)_ = 3.431, *p* < 0.0001; Extended Data Fig. 4-1*E*).

In the NAc shell, repeated-measures analyses also revealed a significant phenotype × current interaction for spike number (*F*_(38,1023)_ = 1.732, *p* = 0.0042) and firing frequency (*F*_(38,1004)_ = 2.135, *p* < 0.0001). Planned pairwise comparisons demonstrated that IR MSNs fired significantly more spikes than STs (*F*_(19,607)_ = 3.451, *p* < 0.0001), whereas GTs did not differ from STs (*p* = 0.6605) or IRs (*p* = 0.2140; Fig. 6*E*). For firing frequency, IRs again exhibited greater excitability, firing at higher rates than both STs (*F*_(19,588)_ = 4.041, *p* < 0.0001) and GTs (*F*_(19,678)_ = 1.797, *p* = 0.0198), while STs and GTs did not differ (*p* = 0.4513; Fig. 6*F*). Together, these results indicate a region-dependent pattern of excitability: in the core, STs are less excitable than both GTs and IRs, whereas in the shell, IRs are the most excitable, with STs and GTs showing broadly similar firing properties.

Beyond the group-level differences, we tested whether PavCA index as a continuous measure was related to neuronal excitability. Exploratory regressions between PavCA index and spike number at 500 pA (Extended Data Fig. 4-2*A*,*D*) showed a modest but nonsignificant negative trend in the core (*R*^2^ = 0.09, *p* = 0.12) and no relationship in the shell (*R*^2^ = 0.02, *p* = 0.47). These analyses should be interpreted cautiously, as the relatively small per-animal sample sizes, the clustering of PavCA scores near the extremes of the index, and the likelihood that excitability–behavior relationships are not strictly linear all limit the utility of simple regressions in this context. Accordingly, while regression results did not reveal robust associations, the group-level findings provide strong evidence that excitability differs categorically across phenotypes.

We also found significant subregional differences in firing properties between the core and shell. Planned comparisons revealed a subregion × current step interaction, suggesting greater firing in the shell than in the core in IRs (*F*_(19,798)_ = 4.653, *p* < 0.0001), a trend in STs (*F*_(19,737)_ = 1.498, *p* = 0.0788), and no difference in GTs (*p* > 0.999). Importantly, per-animal repeated-measures analyses corroborated these subregion × current step interactions, showing significant core–shell differences for both STs (*p* = 0.001) and IRs (*p* < 0.0001), while GTs (*p* = 0.9862) again showed no difference (data from Extended Data Fig. 4-1*E*,*J*). These findings suggest that enhanced shell excitability is most robust in STs and IRs, whereas GTs display comparable spike output across subregions. While the functional significance of these differences remains unclear, they may reflect variations in how each phenotype integrates and responds to motivationally relevant cues.

Lastly, we examined whether specific properties of the action potentials (APs) of MSNs in the core and shell were different between STs, GTs, and IRs. We delivered 5 ms current injections in 25 pA increments until a single AP was elicited and found that the action potential properties in both subregions did not differ between STs and GTs (data not shown in figure; see Table 1 for a full list of AP properties). In summary, no differences were observed in the current required to elicit a single AP, AP half-width, AP threshold from zero and resting potential, or AP peak amplitude from zero, resting potential, and threshold (*p* > 0.05). However, we identified subtle differences between STs and IRs. In the core, STs exhibited a significantly larger AP half-width than IRs (*p* = 0.015), suggesting slower repolarization. In the shell, IRs had a more depolarized AP threshold (*p* = 0.03), and a consequently smaller AP peak amplitude measured from threshold (*p* = 0.027).

**Table 1.** Electrophysiological passive and active properties of medium spiny neurons in the core and shell of nucleus accumbens of STs, GTs, and IRs rats

	Core			Shell		
	Sign-trackers	Goal-trackers	Intermediates	Sign-trackers	Goal-trackers	Intermediates
Passive membrane properties						
Resting membrane potential, mV	−81 ± 1 (23)	−80 ± 1 (17)	−81.5 ± 0.8 (31)	−82.9 ± 0.7 (19)	−79.4 ± 0.9 (23)	−80.6 ± 0.8 (15)
Cell capacitance, pF	150 ± 8 (23)	130 ± 7 (17)	122 ± 5 (31)	105 ± 6 (19)	88 ± 4 (23)	86 ± 6 (15)
Input resistance, MΩ	69 ± 7 (23)	77 ± 7 (17)	71 ± 6 (31)	80 ± 8 (19)	99 ± 7 (23)	97 ± 7 (15)
Active membrane properties						
*V*/*I* curve (−500 to 0 pA)	−11.9 ± 0.2 (19)	−13.6 ± 0.2 (18)	−13.3 ± 0.2 (18)	−14.3 ± 0.3 (15)	−16.4 ± 0.2 (23)	−16.9 ± 0.3 (11)
*V*/*I* curve (0 to +100 pA)	5.3 ± 0.4 (19)	6.2 ± 0.4 (18)	5.6 ± 0.4 (18)	7.7 ± 0.6 (15)	8.4 ± 0.5 (23)	9.8 ± 0.6 (11)
Sag ratio at −500 pA, mV	1.026 ± 0.003 (19)	1.031 ± 0.003 (18)	1.029 ± 0.003 (18)	1.045 ± 0.004 (15)	1.050 ± 0.002 (23)	1.053 ± 0.006 (11)
Number of spikes, AP#	4.7 ± 0.2 (23)	6.4 ± 0.2 (17)	5.7 ± 0.2 (31)	6.5 ± 0.3 (19)	7.0 ± 0.2 (23)	8.3 ± 0.3 (15)
Firing frequency, Hz	10.0 ± 0.4 (23)	13.7 ± 0.4 (17)	12.2 ± 0.3 (31)	14.0 ± 0.5 (19)	14.6 ± 0.5 (23)	17.7 ± 0.6 (15)
Current to threshold, pA	981 ± 56 (20)	875 ± 60 (16)	879 ± 42 (27)	836 ± 63 (19)	711 ± 45 (23)	694 ± 40 (13)
AP threshold, mV	−45 ± 1 (20)	−44 ± 1 (16)	−45 ± 1 (27)	−45 ± 2 (19)*	−41 ± 1 (23)	−39 ± 2 (13)
Δ RMP/AP threshold, mV	36 ± 2 (20)	35 ± 1 (16)	37 ± 8 (27)	37 ± 2 (19)	38 ± 1 (23)	42 ± 2 (13)
AP amplitude, mV	53.9 ± 0.9 (20)	53 ± 1 (16)	51 ± 1 (27)	53 ± 1 (19)	53 ± 2 (23)	49 ± 2 (13)
Δ RMP/AP amplitude, mV	135 ± 1 (20)	132 ± 2 (16)	132 ± 1 (27)	135 ± 1 (19)	133 ± 2 (23)	130 ± 2 (13)
Δ AP threshold/AP amplitude, mV	99 ± 2 (20)	97 ± 2 (16)	96 ± 2 (27)	97 ± 2 (19)*	94 ± 2 (23)	88 ± 3 (13)
AP half-width, ms	0.72 ± 0.02 (20)*	0.68 ± 0.02 (16)	0.65 ± 0.01 (27)	0.70 ± 0.02 (19)	0.68 ± 0.02 (23)	0.69 ± 0.02 (13)

Table lists mean ± SEM (sample size) for passive and active properties of core and shell MSNs for STs, GTs, and IRs. Significance for Tukey's post hoc test between STs and IRs is shown as *p < 0.05.

These findings highlight distinct electrophysiological properties of MSNs in the NAc core and shell across STs, GTs, and IRs, reflecting phenotype-specific differences in intrinsic excitability and firing frequencies.

## Discussion

This is the first study to demonstrate that MSNs in the NAc of STs, GTs, and IRs exhibit distinct intrinsic membrane properties, with differences observed across the core and shell subregions. Most notably, STs had the lowest intrinsic excitability in the NAc core. Although STs and GTs did not differ in the shell firing properties, IRs showed greater intrinsic excitability than both phenotypes. Given that the NAc core is thought to encode the motivational value of conditioned cues, while the shell appears to modulate unconditioned responses (Meredith et al., 2008; Saunders et al., 2018), it is unsurprising that the strongest phenotype differences emerged in the core.

These findings suggest that the previously reported differences in NAc core and shell activity during pavlovian learning may be linked to the intrinsic excitability state of their MSNs. STs show increased cue-evoked activity in both the NAc core and shell (Flagel et al., 2011a), alongside a reduction in reward-evoked activity over PavCA training (Gillis and Morrison, 2019). This pattern, often interpreted as a prediction error signal, is notably absent in GTs despite their clear learning of cue–reward relationship**s**. This has led to the hypothesis that the shift from reward-evoked to cue-evoked activity in the NAc may primarily reflect the attribution of incentive properties to a reward-paired cue as opposed to predictive properties. Here, we found that GTs exhibited higher intrinsic excitability in the NAc core, raising the possibility that a higher neuronal excitability state in GTs may make it more difficult for their incentive salience-related NAc core activity to shift away from the reward and toward the cue. Thus, some of the differences in reward learning processes between GTs and STs might be traced to subregional differences in the intrinsic neurophysiological properties of their NAc core neurons.

Reduced intrinsic excitability of MSNs is a well-established feature of cocaine exposure and withdrawal (for review, see Wolf, 2010). Lower MSN excitability has been directly linked to increased cocaine sensitization and self-administration (Dong et al., 2006; Mu et al., 2010). Behaviorally, STs exhibit greater addiction-like behaviors than GTs, including enhanced psychomotor sensitization to cocaine (Flagel et al., 2008), higher cocaine preference over food (Tunstall and Kearns, 2015), and increased cue-induced reinstatement of cocaine and nicotine (Saunders and Robinson, 2011; Versaggi et al., 2016). Therefore, reduced MSN excitability in STs may be a neurobiological feature that predisposes them to pathological drug responses.

The decrease in MSN excitability following cocaine exposure and withdrawal has been linked to reductions in voltage-dependent Na^+^ (Zhang et al., 1998) and Ca^2+^ (Zhang et al., 2002) currents, as well as increased K^+^ channel activity (Hu et al., 2004). One candidate mechanism is SK-type Ca^2+^-activated K^+^ channels, as blocking these channels with apamin partially prevents cocaine-induced reductions in MSN excitability (Ishikawa et al., 2009). Interestingly, inositol and corticosterone—neurochemicals that differ between STs and GTs (Flagel et al., 2009; Fitzpatrick et al., 2016)—can modulate SK channel activity, increasing K^+^ currents and reducing neuronal excitability (Yamada et al., 2004; Kye et al., 2007; Clements et al., 2013). This raises the possibility that the lower intrinsic excitability observed in STs, even in the absence of drug exposure, may be linked to differences in K+ channel regulation. Future studies should explore whether SK-type K+ channels contribute to phenotype-specific differences in MSN excitability.

It is important to note that most of the mentioned studies reporting a reduction in MSN excitability have been in the NAc shell. While STs and GTs differed in firing properties primarily in the core, ST MSNs in the shell exhibited a significantly more hyperpolarized resting membrane potential, suggesting a lower baseline excitability. Additionally, both STs and GTs showed reduced shell excitability compared with IRs. Few studies have examined IRs in behavioral and neurobiological contexts, making it difficult to predict how their excitability differences relate to behavior. It is known that appetitive pavlovian conditioning can increase the intrinsic excitability of specific neuronal ensembles of MSNs in the NAc shell (Ziminski et al., 2017). It is possible that an overall greater intrinsic excitability in the NAc shell of IRs may facilitate the attribution of both predictive and incentive properties to cues, giving them more flexibility in their conditioned responses. However, additional studies are needed to characterize IRs at this level.

Dopaminergic transmission strongly influences MSN excitability in the NAc (O’Donnell and Grace, 1996; Nicola et al., 2000; Surmeier et al., 2007; Planert et al., 2013). During pavlovian learning, STs and GTs exhibit distinct patterns of dopamine (DA) release, with DA responses mirroring cue- and reward-evoked activity in the NAc (Flagel et al., 2011b). Additionally, DA agonists and antagonists differentially affect STs and GTs (Saunders and Robinson, 2012; Chow et al., 2016), suggesting pre-existing differences in the dopaminergic system. Indeed, some studies have reported differences in the DA system of STs and GTs that may modulate the way MSNs respond to DA and therefore affect their intrinsic membrane excitability properties (Flagel et al., 2007; Singer et al., 2016). For example, STs have greater dopamine transporter (DAT) expression in the NAc core than GTs (Singer et al., 2016), leading to faster DA clearance from the synapse, independent of baseline DA release levels (Singer et al., 2016). This rapid DA reuptake is thought to be important for more time-locked responses of MSNs to phasic DA release during cue presentations and may help potentiate the value of incentive stimuli and attribution of incentive salience (Wieland et al., 2015; Singer et al., 2016). Interestingly, activation of D_1_-like receptors in MSNs can cause an increase in MSN depolarization by inhibiting Kir-channel K^+^ currents (Podda et al., 2010) and by enhancing L-type Ca^2+^ currents (Hernández-López et al., 1997). Therefore, if GTs have greater tonic DA levels in the NAc, this may lead to stronger D1 receptor activation increasing membrane excitability compared with STs. Lower intrinsic excitability in STs, combined with a more fine-tuned response to DA release, may selectively enhance the response of MSNs to only the most salient stimuli and thereby promote the attribution of incentive salience to specific stimuli. Additionally, D_1_-mediated excitability occurs mainly when MSNs are already near threshold (up-state), which may explain why GT MSNs exhibit higher excitability in depolarized but not hyperpolarized states.

We found that subregional differences between core and shell MSNs were present for STs and IRs, but not for GTs. In both STs and IRs, MSNs in the shell fired more action potentials than those in the core, whereas GTs showed no significant subregional difference. It is interesting that IRs share this feature with STs. In this case, this shared characteristic may suggest that a subregional difference might be important for sign-tracking responses. Future studies are needed to test whether experimental manipulation of subregional differences between core and shell excitability can affect individual differences in the attribution of incentive salience.

A key limitation of this study is the inability to determine whether MSN excitability differences in STs and GTs are innate or shaped by PavCA training. This is because currently, there is no method to distinguish STs from GTs before training, making it unclear whether observed excitability differences are pre-existing or experience dependent. First, the training experience itself (e.g., handling, reward exposure) may be enough to alter NAc physiology (Scala et al., 2018) and second, the actual learning component (e.g., CS–US associations) of the task may also have a distinct impact (Ziminski et al., 2017). We attempted to control for these variables by waiting a period of at least 1 week before the electrophysiological recordings with the hopes that any training-induced changes would most likely have returned to baseline by then. Based on this limitation, although we found clear differences between STs, GTs, and IRs in membrane properties of MSNs, we could not determine whether these are pre-existent differences and conclude that all three phenotypes are innately different. To determine whether individual differences in PavCA behavior are innate and caused by intrinsic differences in NAc excitability, some method would be needed to identify STs and GTs prior to PavCA training, and/or measurement of MSN excitability would have to be done in vivo to test how excitability changes over the course of behavioral training.

Another important limitation is that these studies did not include female rats. The study design necessitated a comparison of STs, GTs, and IRs, and the testing conditions employed resulted in an acceptable distribution of behavioral phenotypes only in the males. Thus, it remains unclear whether the current findings would extend to both sexes. Future studies optimized for testing female rats or for examining sex differences are needed to answer that question.

The overwhelming evidence suggests that neuronal activity in the NAc is particularly important for individual differences in the attribution of incentive salience. It is very likely that the distinct patterns of activity during PavCA are the collective result of differences in inputs to the NAc modulated by both internal and external stimuli in STs and GTs. However, our results suggest that these characteristic patterns of activity can be directly influenced by differences in the intrinsic neuronal properties of MSNs in the NAc of STs and GTs. This in turn may help us understand how inputs to the NAc may exert different synaptic influence because of baseline differences in its excitability state. We intend for these studies to aid in our understanding on the neurobiology of individual differences in incentive salience attribution and endophenotypes associated with addiction vulnerability.

## Synthesis

Reviewing Editor: Christie Fowler, University of California Irvine Decisions are customarily a result of the Reviewing Editor and the peer reviewers coming together and discussing their recommendations until a consensus is reached. When revisions are invited, a fact-based synthesis statement explaining their decision and outlining what is needed to prepare a revision will be listed below. The following reviewer(s) agreed to reveal their identity: Brandon Henderson. Note: If this manuscript was transferred from JNeurosci and a decision was made to accept the manuscript without peer review, a synthesis may not be available.

The authors employed a Pavlovian conditioning procedure to separate a cohort of 31 outbred rats into those that interact more with a lever cue that predicts reward (sign-trackers) vs those that interact little with the same cue (goal-trackers). A third intermediate group captured the rats in between. One to three weeks later the brains were extracted for acute slice physiology recordings from NAc MSNs, including shell and core sub-regions, to compare intrinsic properties between these behaviorally defined groups across a range of variables. Many similarities were observed between the groups, as well as a few differences that generally suggest somewhat less excitability in NAc MSNs of sign trackers. The work is well done, the methods and results are clearly described, and the results are reasonably interpreted, including the appropriate identification of the key limitation (i.e., whether differences were pre-existing or induced by the conditioning). It was also noted that the strengths of this work are the use of an operant behavior paradigm and the integration of whole-cell patch-clamp electrophysiology. In the reviewer consultation session, the main points discussed included the need to show the data for how each subject/group did or did not differ with respect to the gap between behavior and brain extraction, any variable measured repeatedly from the same subject/cell should include a repeated measures dimension, and regression analyses should be included to complement the posthoc group/split. The specific integrated reviewer comments are as follows:

Reviewer Comments:

1. I'm not convinced it is necessary or appropriate to apply statistics on data presented in figures 2 and 3. Any dataset that is split by a continuous dependent variable/s and then compared for that same dependent varaible/s using statistical methods will show differences if sufficiently powered. But a core assumption of statistical methods is that the data are from randomly sampled populations, by grouping in this way that assumption is broken. The recommendation is to remove the parametric statistical comparisons, graphs would stay, but treated as the descriptive statistics that they are.

2. I have no particular concerns about grouping a continuous variable in one category (tracking behavior) and then doing statistical comparisons across other independently measured parameters, as done for all the electrophysiological measurements. However, I generally think a regression-based analysis has more utility and power, because it need not impose the arbitrary cut-offs needed for grouping. And because it doesn't throw away data, for example by lumping together the intermediate trackers that at their extremes are much more like sign-trackers or goal-trackers than they are like each other. Did/can the authors complement the current analyses by including regression analyses that compare the conditioning index on one axis with each of the physiological measurements on the other, adding these to their respective figures?

3. The authors prepare brain slices for patch-clamp electrophysiology over a range of 1 - 3 weeks after the end of the PavCA training. The authors do not provide any specific details if the prepared slices from the ST, GT, and IR groups had similar or identical timing for the recovery. This could present a major problem with the interpretation of the data. Since the slices are listed as simply 1-3 weeks, without any indication of group, it is possible that one group had 1 week recovery and another group had 3 weeks of recovery.

4. The difference in recovery itself could trigger changes in physiological properties that could create a group difference. If the authors could provide clear details of the timeline of recovery for each group, that would be sufficient. Differences in recovery time will result in differences with intrinsic excitability.

5. Moreover, the range of 1-3 weeks as an experimental design is perplexing. Why not have all the recordings done at 3 weeks (or 2 or 1)? This could be done with staggering the start and stop of the PavCA paradigm so that all of the recordings have the same time point.

6. The electrophysiological data in Figures 4 and 5 appear to be values from individual cells. Because this data is being recorded from the same animals that completed the behavior assays, the authors should consider just using a mean value for each individual animal.

Minor comments:

1. Given the hypothesis of differences in K+ currents, the authors could get some insight into this by calculating differences in the anti-peak of their action potentials

## Author Response

Response to Reviewers We thank the reviewers and the editor for their thoughtful and constructive feedback. Below we provide a point-by-point response, with reviewer comments in italics followed by our responses. All manuscript changes are indicated in the revised text, with updates in the Methods, Results, and Discussion as noted.

A. Statistical Analyses 1. Figures 2 &3 - Remove inappropriate stats "Statistical tests on Figures 2 and 3 are not appropriate, since the same dependent variable was used to define the groups." Response:

We thank the reviewer for raising this important point. We have removed the parametric statistical comparisons from Figures 2 and 3 and now present these data descriptively (means {plus minus} SEM). The graphs remain to illustrate the clear divergence of sign-tracker, goal-tracker, and intermediate phenotypes during Pavlovian conditioning. The corresponding figures and text (Methods, Results, and Figure Legends) have been revised to describe the data without statistical inference.

2. Regression analyses (Reviewer request) "Consider regression analyses between PavCA index (continuous measure) and electrophysiological variables, rather than relying solely on categorical grouping." Response:

We thank the reviewer for this helpful suggestion. We have now conducted exploratory regression analyses between PavCA index (Sessions 5-6 mean) and intrinsic membrane properties, performed separately for NAc core and shell. For each animal, electrophysiological values were averaged across recorded cells to yield one data point per region, and linear regressions were run in GraphPad Prism (Supplementary Figure S1). We focused on resting membrane potential (RMP), input resistance (Rin), and spike number at 500 pA as representative passive and active measures.

These analyses revealed no significant associations in the core, although spike number at 500 pA showed a modest, non-significant negative trend (R² = 0.09, p = 0.115). In the shell, PavCA index was negatively associated with RMP (R² = 0.22, p = 0.015), consistent with the more hyperpolarized RMP observed in STs in our group-level ANOVA analyses. Input resistance did not correlate with PavCA index in either region.

We present these analyses as exploratory, given the limited per-animal sample size, clustering of PavCA scores at the extremes, and the likelihood that excitability-behavior relationships are not strictly linear. Nevertheless, these results are included for completeness and provide convergent support for the group-level differences.

3. Repeated measures dimension "Any variable measured repeatedly from the same subject/cell should include a repeated measures dimension." Response:

We appreciate this important point. In the revised manuscript, we have re-analyzed all electrophysiological measures collected across current injection steps (number of spikes, firing frequency, and V/I curves) using repeated-measures mixed-effects models (REML). These models included current injection as the within-subjects factor and phenotype as the between-subjects factor, with the phenotype × current interaction explicitly tested.

Planned pairwise comparisons (ST vs GT, ST vs IR, GT vs IR) are now reported alongside the overall results. These revisions ensure that repeated-measures structure is properly accounted for and that our conclusions are robust.

B. Experimental Design Clarification 1. Timing of slice recovery (1-3 weeks issue) "Please clarify the recovery interval across phenotypes and justify the 1-3 week range." Response:

We appreciate this concern. Recordings were performed between 1 and 3 weeks following PavCA training, with all phenotypes represented across this interval and no systematic differences in recovery times between groups (Supplementary Table S1). This resting period was intentional, to allow acute training-induced plasticity to subside so that electrophysiological measurements reflected baseline intrinsic properties rather than short-term adaptations.

The distribution of recovery times was balanced across phenotypes (median = 7.5 days for all groups), minimizing the likelihood that interval length confounded our results. We have clarified this in the Methods and now provide a summary of the recovery-time distribution in Supplementary Table S1.

C. Additional Analyses &Clarifications 1. Mean per animal (Figures 4-6) "Consider re-plotting data with each dot = animal mean, not cell." Response:

We thank the reviewer for this thoughtful suggestion. In addition to our primary cell-level analyses, we computed per-animal mean values by averaging across all recorded cells within each region for each rat. These data are now presented in Supplementary Figure S2.

At the per-animal level, overall group ANOVAs across STs, GTs, and IRs were not significant. However, planned comparisons focusing on the ST vs GT contrast revealed convergent effects: STs exhibited more hyperpolarized RMP in the shell than GTs, and repeated-measures analyses showed a significant ST vs GT interaction in the core for spike number across current steps. These findings are consistent with our cell-level and regression results. We therefore retain the cell-level plots in the main figures to illustrate within-animal variability, while providing per-animal analyses in the Supplement to demonstrate robustness across analytic approaches.

2. K⁺ currents / AP anti-peak (minor) "Consider reporting AP anti-peak or K⁺ current measures, if available." Response:

We appreciate this suggestion. AP anti-peak values were not extracted from the present dataset. However, we agree that differences in K⁺ channel activity represent a compelling candidate mechanism for phenotype-specific reductions in MSN excitability. We highlight this in the Discussion, citing prior work on Na⁺, Ca²⁺, and K⁺ currents in MSNs (Zhang et al., 1998; Hu et al., 2004; Ishikawa et al., 2009). We also propose SK-type Ca²⁺-activated K⁺ channels as a future direction for understanding the reduced excitability observed in STs.

We thank the reviewers again for their valuable feedback, which has led to substantial improvements in the rigor, clarity, and interpretability of the manuscript.
