# Supplementary material for: Individual Variation in Intrinsic Neuronal Properties of Nucleus Accumbens Core and Shell Medium Spiny Neurons in Male Rats Prone to Sign- or Goal-Track
Source: eNeuro. 2025 Dec 12;12(12):ENEURO.0203-25.2025. doi: 10.1523/ENEURO.0203-25.2025 (PMC12705225; doi:10.1523/ENEURO.0203-25.2025)
Supplement: Figure 1-1 — Distribution of recovery times across phenotypes. Days post-PavCA training to slice preparation are shown for each phenotype. The intended design was a 7-day recovery period; however, because behavioral cohorts were staggered so that each day a rat was ready for recordings, some animals (8 of 31) were recorded beyond 7 days due to equipment availability and scheduling constraints. All phenotypes were represented across the 1–3 week recovery period. Download Figure 1-1, DOCX file. [file eneuro-12-ENEURO.0203-25.2025-s002.docx]

|  | Days post-PavCA training | | |
| --- | --- | --- | --- |
| Phenotypes | **7-8 days** | **10-12 days** | **19-25 days** |
| ST (n = 10) | 6 | 2 | 2 |
| GT (n = 7) | 6 | 0 | 1 |
| IR (n = 14) | 11 | 1 | 2 |
| All (n = 31) | 23 | 3 | 5 |
